# Particle-Attached and Free-Living Archaeal Communities in the Benthic Boundary Layer of the Mariana Trench

**DOI:** 10.3389/fmicb.2018.02821

**Published:** 2018-11-21

**Authors:** Hongmei Jing, Wenda Zhu, Hongbin Liu, Liping Zheng, Yu Zhang

**Affiliations:** ^1^CAS Key Lab for Experimental Study Under Deep-sea Extreme Conditions, Institute of Deep-sea Science and Engineering, Chinese Academy of Sciences, Sanya, China; ^2^University of Chinese Academy of Sciences, Beijing, China; ^3^Division of Life Science, The Hong Kong University of Science and Technology, Kowloon, China; ^4^State Key Laboratory of Ocean Engineering, Institute of Oceanography, Shanghai Jiao Tong University, Shanghai, China

**Keywords:** benthic boundary layer, archaea, Mariana Trench, particle-attached, free-living

## Abstract

The benthic boundary layer (BBL) is the part of the water column that is situated near to the sediment surface, where active oceanic biogeochemical cycling occurs. Archaea play an important role in mediating this cycling, however, their composition and diversity in the BBL remain largely unknown. We investigated the community composition and abundance of both particle-attached (PA) and free-living (FL) archaea in the BBL on the slopes of the Mariana Trench using Illumina sequencing and quantitative PCR (qPCR), at both the DNA and RNA levels. Our results showed that Thaumarchaeota (>90%) and Woesearchaeota (1–10%) dominated in all the BBL samples, and that the former was composed mainly of Marine Group I (MGI). A clear separation of PA and FL samples was observed, and they showed a high level of similarity to the subsurface sediments and the water column, respectively. No significant differences were detected in the archaeal communities located in the southern and northern slopes of the Mariana Trench, or between the levels of DNA and RNA. However, lower RNA/DNA ratios (estimated by qPCR) were found in the PA samples than in the FL samples, indicating higher transcriptional activities in the FL fractions. A distinct archaeal community structure was found in the middle of the trench when compared with samples collected at the same depth at other stations along the trench slopes. This indicates that a dynamic deep current might affect the distribution of organic matter on the slopes. Our study provides direct information regarding the archaeal communities in the BBL of the Mariana Trench. We suggest that this might promote further exploration of the ecological roles and microbial processes of such communities located in deep-sea ecosystems.

## Introduction

The benthic boundary layer (BBL) is defined as the bottom layer of the water column, which lies directly adjacent to the seabed; it is composed of different sub-layers, which range in depth from several meters to just a few millimeters (Turley, 2000; Chong et al., 2017). In shallow estuarine ecosystems, various biogeochemical processes have been detected in the BBL (Duren and Middelburg, 2001; Percuoco et al., 2015; Topping et al., 2016), where nutrients are released from the sediment into the water column (Percuoco et al., 2015; Topping et al., 2016). Although research into the deep-sea BBL has attracted much attention (Turley, 2000; Dell'Anno and Danovaro, 2005; Danovaro et al., 2014; Tarn et al., 2016), studies have up until recently been limited by instrumentation constraints. However, due to significant advancements in the development of deep-sea sample acquisition equipment over the last few years, accumulating research has revealed that even sediments in the deepest ocean can supply nutrients to the bottom water (Glud et al., 2013; Tarn et al., 2016). Moreover, recent studies suggested that the abundance of archaea in the deep-sea BBL is significantly affected by the incorporation rates of carbon fixation in this zone (Dang et al., 2013; Molari et al., 2013). Indeed, one phylum of archaea, Thaumarchaeota, has been suggested to play a pivotal role in the carbon, nitrogen, and phosphorus cycles in marine sediments, especially in the subsurface sediments (Dang et al., 2013). However, environmental factors that might affect the diversity and distribution of Thaumarchaeota in the deep-sea BBL remain unclear.

Most of the previous studies conducted on the deep-sea archaea have been confined to deep sediments, such as hydrothermal vents (Takai and Horikoshi, 1999; Taira et al., 2004), and cold seeps (Li et al., 1999; Zhang and Fang, 2006; Heijs et al., 2008). In these locations, the abundance of archaea was found to range between 10^4^ and 10^8^ cells cm^−3^ (Durbin and Teske, 2012), and Thaumarchaeota were shown to dominate the archaeal community in almost all deep-sea subsurface cases (Tully et al., 2012). Nutrients (mainly ammonia and sulfide) and particulate organic matter (POM) have been recognized as the predominant factors that influence the abundance and activity of microbes in these regions (Durbin and Teske, 2012; Moeseneder et al., 2012; Nunoura et al., 2013). In addition, distinct archaeal lineages that are rare in the euphotic layer (such as Marine Group I, Marine Benthic Group A and most of the Deep-sea Hydrothermal Vent Euryarchaeotal Group), have been found in the sediment of the oligotrophic ocean (Durbin and Teske, 2012). Most of the water that over-lies deep-sea sediments has been shown to present similar archaeal community structure to those in subsurface sediments (Durbin and Teske, 2011, 2012). In addition, the availability of organic matter is recognized as being the most important factor in determining the community structure of archaea in both the sediment and in the thin overlaying sediment-water interface (Durbin and Teske, 2012; Dang et al., 2013; Nunoura et al., 2013). Although the Challenger Deep and the Sirena Deep regions of the Mariana Trench share very similar physical and chemical parameters, they have different archaeal community compositions in the BBL (Tarn et al., 2016). This suggests that other factors must influence the archaeal communities in the deep-sea BBL, such as the amount and lability of the particular organic matter (POM) that is received from the upper layers.

The BBL contains a high concentration of particles that are resuspended from the subsurface sediments (Turley, 2000). Although the size fractions of bacteria with distinct taxonomic compositions have been identified (DeLong et al., 1993; Hollibaugh et al., 2000; Smith et al., 2013; Suter et al., 2018), the difference regarding archaeal community structure between particle-attached (PA) and free-living (FL) fractions is still largely unknown. Recently, several metatranscriptomic studies have reported unequal transcript profiles for the PA and FL archaea (Ganesh et al., 2015; Satinsky et al., 2017), but any differences between the PA and FL archaeal communities were not reported. In addition, most studies focusing on the size of archaea were conducted only at the DNA level. However, Ganesh et al. (2015) reported that some N-cycling microbes (such as ammonia-oxidizing archaea) have higher RNA/DNA ratios in the FL fraction than in the PA fraction. All of the above observations suggest that a comprehensive evaluation of the microbial communities in the PA and FL fractions might be obtained by conducting molecular studies on size-fractioned prokaryotes at both the DNA and RNA level.

Trenches are the deepest oceanic areas on Earth and they are characterized by a low temperature and an extremely high hydrostatic pressure. In several studies, endemic microorganisms have been found living in trenches (Jamieson et al., 2010; Danovaro et al., 2014; Tarn et al., 2016). Indeed, archaea have been discovered in the Ogasawara and Mariana Trenches, and their community composition has been shown to shift along the trench vertical profile (Nunoura et al., 2013, 2016). In the Mariana Trench, for example, a higher proportion of heterotrophic archaea were found in the deepest water than in the shallower waters (Nunoura et al., 2016; Tarn et al., 2016). However, in general, research to identify the archaea living along trench slopes remains scarce. Similar to other abyssal locations, the northern and southern slopes of the Mariana Trench show similar physical and chemical conditions, such as salinity, temperature, and oxygen concentration (Taira et al., 2004, 2005). However, the southern slopes of the Mariana Trench are subject to more perturbation due to frequent submarine earthquakes (Heeszel et al., 2008; Chen et al., 2015), and so they show stable but low concentrations of organic compounds across all the depths (Luo et al., 2017). Investigating the southern slopes of the Mariana Trench might therefore help to elucidate whether a shared microbial community exists for the archaeal assemblages and identify the potential shaping forces of the deep-sea archaeal communities.

In this study, we focused on the archaeal communities in the BBL on the northern and southern slopes of the Mariana Trench. We performed pyrosequencing at both the DNA and RNA levels to investigate the composition of the FL and PA archaeal communities in these locations, and we explored the environmental factors that might control archaeal diversity and activity in this biogeochemically important interface between the water and sediment in the deep ocean.

## Materials and methods

### Sample collection

**Figure 1:**
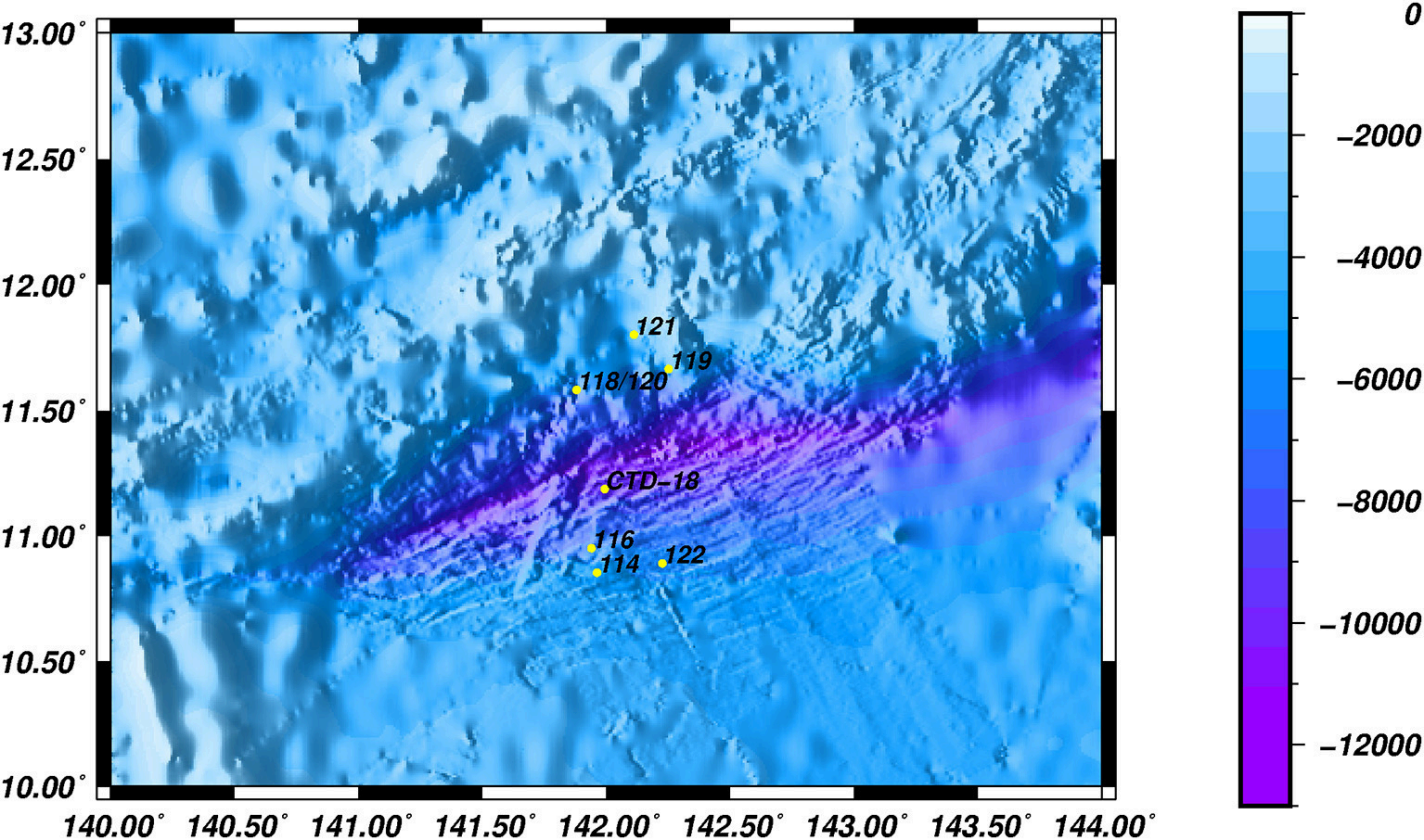
The locations of the various sampling stations.

A total of 8 stations, with water depths ranging from 5,482 to 6,697 m, were investigated during a cruise in June 2016. Water samples were collected in Niskin bottles from the southern (Dive-114/116/122), middle (CTD-18) and northern (Dive-118/119/120/121) regions of the Mariana Trench in the Western Pacific Ocean (Figure 1). Samples were collected either by the Jiao Long Human Occupied Vehicle (HOV) or by CTD cassette, and to differentiate between these two collection methods, the samples were labeled Dive-114 to Dive-122, or CTD-18, respectively. CTD-18 was collected from the central region of the trench far from the benthic sediment, and this was used as a control. Except for Dive-114, which was collected at ~5 m above the sediment surface, all of the other Dive samples were collected tens of centimeters above the sediment surface once the Jiao Long HOV had landed.

For DNA/RNA collection, 2–5-liter water samples were sequentially filtered through 3.0 and 0.2 μm polycarbonate filters (47 mm, EMD Millipore, Billerica, MA, USA). All the filters were stored in RNA*later*™ Stabilization Solution (Thermo Scientific, Wilmington, DE, USA) and stored at −80°C until required for further analysis. *In situ* hydrographical parameters (i.e., temperature and salinity) were recorded by a conductivity-temperature-depth (CTD) rosette system (Sea-Bird Electronics Inc., Bellevue, WA, USA), while the concentration of nutrients (e.g., ammonia, silicon, and phosphorus) were measured with an auto-analyser (QuAAtro, Blue Tech Co., Ltd., Tokyo, Japan).

### DNA and RNA extraction and cDNA synthesis

Total DNA was extracted with a PureLink Genomic DNA kit (Invitrogen, Carlsbad, CA, USA) according to the manufacturer's protocol, and the total RNA was extracted with TRIzol® Reagent and an RNA purification kit (Invitrogen), again according to the manufacturer's instructions. The concentrations of DNA and RNA were determined with a NanoDrop 2000C spectrophotometer (Thermo Scientific, Wilmington, DE, USA). RNA was purified with DNase I (Ambion, Life Technologies, Austin, TX, USA), and then reverse transcribed with a SuperScript III First-strand Synthesis kit (Invitrogen). A parallel reaction (but without SuperScript III RT), was used as a negative control (non-RT control) for the RT-PCR conducted for each sample. Residual RNA was removed by treatment with 2 U RNase H at 37°C for 20 min. DNA and cDNA were stored at −20°C prior to further analysis.

### DNA and cDNA amplification and pyrosequencing

The DNA and cDNA were amplified by targeting the archaeal V3-V4 regions of the 16S rRNA gene using the FastStart High Fidelity PCR system (Roche) with the following primer pair: 344F (5′-ACGGGGYGCAGCAGGCGCGA-3′) (Raskin et al., 1994) and 806R (5′-GGACTACVSGGGTATCTAAT-3′) (Takai and Horikoshi, 2000). Touch-down PCR was performed with the following conditions: an initial denaturation step at 95°C for 3 min, then 35 cycles of annealing beginning at 63°C and ending at 54°C for 30 s, and finally an extension step at 72°C for 30 s. The annealing temperature was decreased by 1°C/cycle until it reached 55°C. A negative control was also included during amplification in order to detect potential contamination. Amplification and paired-end sequencing of the amplicons were performed with an Illumina MiSeq sequencer (BGI Co., Ltd.; http://www.genomics.cn).

### Quantitative PCR

The archaeal 16S rRNA gene and gene transcripts were quantified via a StepOnePlus Real-Time PCR System (Applied Biosystems Inc., Carlsbad, CA, USA), with the same primers that were used in the amplification process. Reaction mixtures contained: 10 μL 2 × SYBR® Premix Ex Taq II (TaKaRa Bio Inc., Shiga, Japan), along with each primer at 0.3 μM, 2 μL DNA/cDNA as the template, 0.4 μL ROX (reference dye), and water to a total volume of 20 μL. Quantitative PCR was conducted at the following conditions: an initial denaturation step at 95°C for 1 min, and then 40 cycles of denaturation at 95°C for 10 s, annealing at 60°C for 30 s, and finally an extension step at 72°C for 30 s. Steps were then conducted at 95°C for 30 s, 60°C for 1 min and 95°C for 30 s to determine the melting curve. qPCR was performed in triplicate for each sample, with an efficiency range of ~90–95%, and the gene copy number was normalized to the quantity of the gene and gene transcripts. As a positive control, a linear plasmid was used, which was constructed using the amplified PCR products and a TOPO-TA vector cloning kit (Invitrogen).

### Bioinformatics analysis

The sequencing adaptor and barcodes were removed, and the sequences were de-noised with Mothur (Schloss et al., 2009) after pyrosequencing. A similarity of 97% was used as a cut-off value for defining OTUs (operational taxonomic units). To evaluate the number of shared OTUs among samples, a Venn diagram was generated from the normalized OTU data using the VennDiagram package of R (version 3.4.2). Diversity indices used for comparing the relative complexity of samples of archaea (ACE, Chao1, Simpson and Shannon), were calculated based on the OTU data. The partial archaeal 16S rRNA gene was identified based on SILVA release 128 (Quast et al., 2013) to the genus level.

A phylogenetic tree was constructed to identify the phylogenetic affiliations of the archaeal 16S rRNA gene sequences. Representative sequences of the top 40 OTUs (covering 95% of the total OTUs) were blasted using the nucleotide BLAST (BLASTn) webpage of the National Center for Biotechnology Information (NCBI) nucleotide sequence database (http://blast.ncbi.nlm.nih.gov/Blast.cgi), and several of the most similar sequences among the representative sequences of the top OTUs were selected. The representative sequences, the selected sequences and the environmental sequences of the archaeal 16S rRNA gene from the NCBI database were used to construct a maximum likelihood (ML) tree with PhyML3.0 (Guindon et al., 2010). A model test was conducted using PhyML-SMS (Lefort et al., 2017) to select the best-fit DNA distribution model for construction of the ML tree, after which the K80 model was selected. In addition, a discrete Gamma distribution was used to model the evolutionary rate differences among the sites (+G). The rate variation model allowed for some sites to be evolutionarily invariable (+I). The ML tree was further edited with iTOL (Letunic and Bork, 2016).

### Statistical analyses

To assess a group pattern among samples, a Newick-formatted unweighted pair method with arithmetic mean (UPGMA) tree was generated and non-metric multidimensional scaling (NMDS) analysis was performed using the tree.shared and nmds commands in Mothur. Then, ANOSIM was performed to test the significance of community differences among multiple groups. Redundancy analysis (RDA) was performed with CANOCO V5.0, to estimate correlations between the distributions of archaeal phylogenetic groups and environmental variables. The phylogenetic group data were Hellinger transformed, environmental variables were logarithm transformed, and the effects of collinearity (VIF > 20) were removed. The statistical significance of an explanatory variable added in the course of forward selection was tested with the Monte Carlo permutation test (9,999 permutations, *p* < 0.05). A paired-samples *T*-test was used to test for significant differences between gene and transcript abundances with R (version 3.4.2).

### Nucleotide sequence accession numbers

All of the archaeal 16S rRNA gene and gene transcript sequences retrieved in this study have been deposited in the NCBI Sequence Read Archive (SRA) under accession number SRP145327.

## Results

### Environmental parameters of sampling stations

In general, the temperature (1.6 ± 0.1°C) and salinity (34.60 ± 0.1 psu) were similar among all the stations (Table 1). Higher nutrient concentrations were found at the southern stations than at the northern regions. For example, the lowest nutrient concentrations (i.e., of NH${}_{4}^{+}$, PO${}_{4}^{3-}$, NO${}_{2}^{-}$ and NO${}_{3}^{-}$) were detected in Dive-118, collected in the northern region. The concentrations of NO${}_{2}^{-}$ and NO${}_{3}^{-}$ showed significant correlations with PO${}_{4}^{3-}$ concentration at all stations (*R*^2^ = 0.904, *p* < 0.01).

**Table 1:** Environmental characteristics of the water samples.

**Station**	**Area**	**Longitude**	**Latitude**	**Depth**	**Temperature**	**Salinity**	**NO_2_ + NO${}_{3}^{-}$**	**PO${}_{4}^{3-}$**	**Si**	**NH${}_{4}^{+}$**
		**(^°^E)**	**(^°^N)**	**(mbsf)**	**(^°^C)**	**(psu)**	**(μg/L)**	**(μg/L)**	**(μg/L)**	**(μg/L)**
Dive 114	South	141.9626°	10.8526°	5,482	1.52	34.60	208.3	92.84	1,400	47.83
Dive 122	South	142.2260°	10.8895°	6,300	1.63	34.67	179.3	67.66	1,116	38.83
Dive 116	South	141.9400°	10.9503°	6,501	1.66	34.67	207.7	82.62	1,724	50.51
Dive 118	North	141.8802°	11.5814°	6,682	1.69	34.67	113.9	20.45	911.5	31.16
Dive 119	North	142.2516°	11.6639°	6,002	1.59	34.69	213.7	82.71	1,651	101.31
Dive 120	North	141.8802°	11.5814°	6,697	1.69	34.67	190.1	78.47	1,089	17.33
Dive 121	North	142.1115°	11.8004°	5,577	1.54	34.67	158.7	53.08	899.2	47.68
CTD-18	Middle	141.9931°	11.1861°	5,900	1.58	34.69	210.5	71.31	1,509	37.99

### Abundance and diversity of archaea

**Figure 2:**
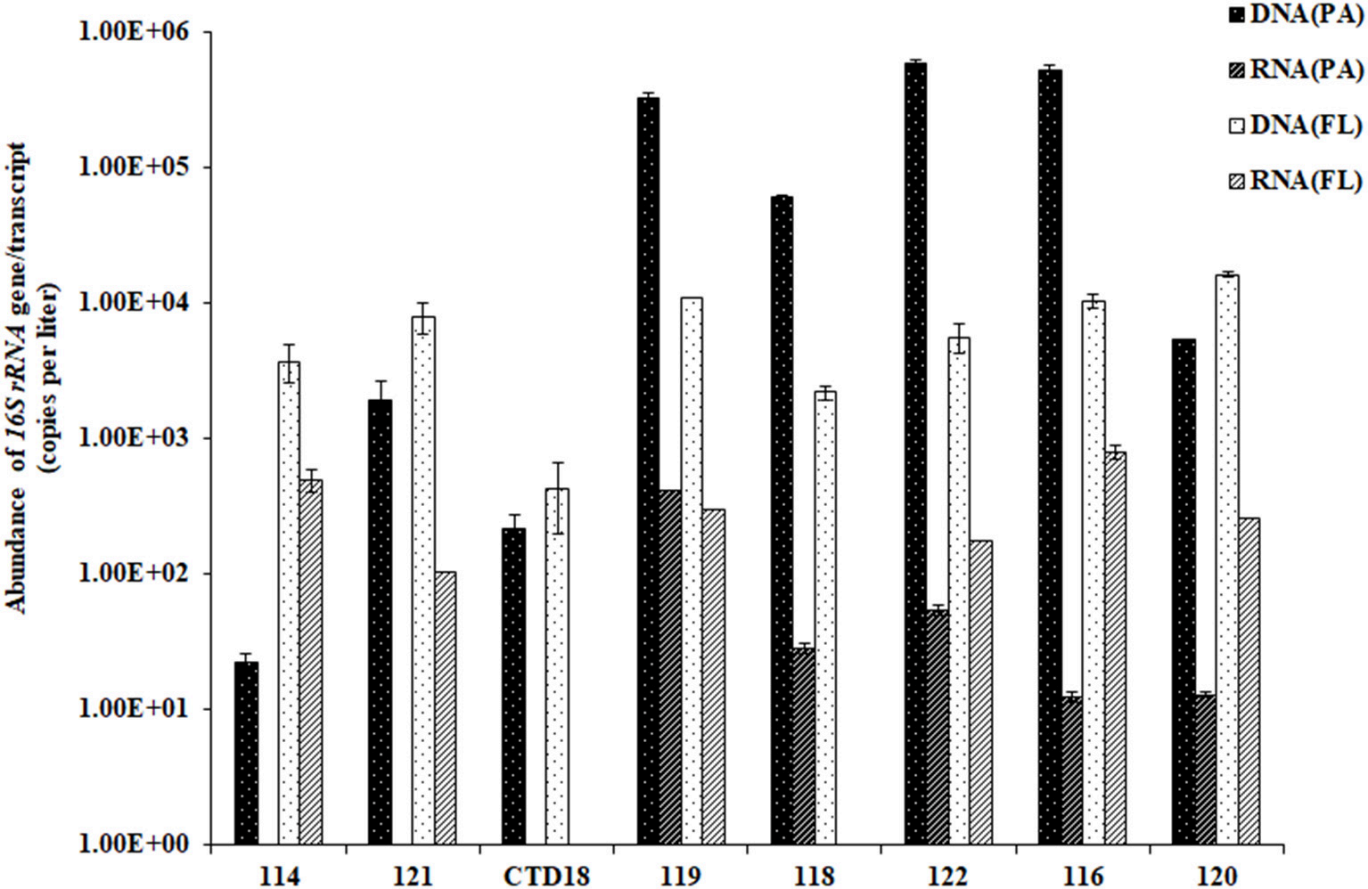
The abundances of the archaeal 16S rRNA gene sequences quantified by qPCR. Some of the RNA (PA) data were below the qPCR detection limit and are therefore not shown in the figure.

The abundances of archaeal 16S rRNA genes ranged from 10 to 50,000 copies per liter in the PA fraction, with ~50–150 copies per liter transcribed. In comparison, the FL fraction had significantly lower archaeal gene abundances (*p* < 0.05) and significantly higher gene transcript abundances (*p* < 0.01) than the PA fraction (Figure 2). In general, however, the archaeal 16S rRNA gene was more abundant than the gene transcript in all of the samples (*p* < 0.05). The ratio of RNA/DNA was higher in the FL dataset than in the PA dataset (*p* < 0.01).

High-throughput pyrosequencing generated ~1.30 million high-quality reads from all 31 samples and the reads were assigned to 2,463 OTUs. For the purpose of comparison, all the samples were normalized to 29,500 reads per sample. There were 266 OTUs shared among all of the datasets (i.e., comprising PA-DNA, PA-RNA, FL-DNA, and FL-RNA; Figure S1), which constituted 24–36% of all of the OTUs in each dataset. The number of OTUs was higher at the DNA level than at the RNA level (*p* < 0.05; Tables 2, 3), but a similar diversity was observed at the DNA and RNA levels and between the PA and FL communities in all of the samples apart from in Dive-114, Dive-119 and CTD-18. In the PA fraction, Dive-119 had a higher Shannon index value in the DNA dataset than in the RNA dataset (Table 2), whereas Dive-114 and CTD-18 had higher diversity values in the RNA dataset than in the DNA dataset.

**Table 2:** Sequencing information and diversity parameters of particle-associated (PA) archaea in the benthic boundary layers of the Mariana Trench.

**Sample**	**Total reads**	**Quality reads**	**OTUs**	**Chao**	**ACE**	**Shannon**	**Simpson**	**Coverage**
			**97%**	**97%**	**97%**	**97%**	**97%**	**97%**
114-PA-cDNA	54,024	33,411	179	297.61	408.11	2.40	0.13	0.99
CTD18-PA-cDNA	53,263	41,524	136	222.77	242.14	1.54	0.40	0.99
116-PA-cDNA	53,064	46,373	163	247.18	291.66	2.29	0.15	0.99
118-PA-cDNA	52,946	33,505	142	195.04	208.79	2.30	0.14	0.99
119-PA-cDNA	53,116	49,886	61	100.00	145.78	0.28	0.91	0.99
120-PA-cDNA	53,295	48,443	294	373.92	372.91	2.63	0.11	0.99
121-PA-cDNA	52,846	39,748	73	113.62	165.35	1.84	0.19	0.99
122-PA-cDNA	53,985	44,203	251	359.37	396.33	2.49	0.15	0.99
114-PA-DNA	53,745	40,739	38	84.20	146.14	0.83	0.48	0.99
CTD18-PA-DNA	53,371	38,091	117	152.15	189.34	0.69	0.75	0.99
116-PA-DNA	53,924	40,344	387	754.60	186.19	2.25	0.18	0.99
118-PA-DNA	53,242	46,195	356	517.63	533.94	2.42	0.13	0.99
119-PA-DNA	53,547	35,435	459	679.66	960.29	2.54	0.15	0.99
120-PA-DNA	53,737	29,586	251	363.86	425.74	2.96	0.11	0.99
121-PA-DNA	53,331	48,671	248	329.62	314.33	2.61	0.11	0.99
122-PA-DNA	54,011	35,987	452	676.01	863.87	2.53	0.15	0.99

**Table 3:** Sequencing information and diversity parameters of free-living (FL) archaea in the benthic boundary layers of the Mariana Trench.

**Sample**	**Total reads**	**Quality reads**	**OTUs**	**Chao**	**ACE**	**Shannon**	**Simpson**	**Coverage**
			**97%**	**97%**	**97%**	**97%**	**97%**	**97%**
114-FL-cDNA	53,314	39,473	176	251.14	242.98	1.32	0.43	0.99
CTD18-FL-cDNA	55,252	33,463	131	195.69	182.29	0.81	0.68	0.99
116-FL-cDNA	54,603	48,334	412	492.73	499.98	1.79	0.37	0.99
119-FL-cDNA	54,220	41,069	339	449.86	416.43	2.11	0.25	0.99
120-FL-cDNA	54,524	31,778	274	358.09	351.36	3.01	0.11	0.99
121-FL-cDNA	53,868	46,974	214	249.65	240.99	1.71	0.36	0.99
122-FL-cDNA	54,889	38,897	179	333.17	357.75	2.23	0.20	0.99
114-FL-DNA	53,343	49,210	167	264.24	286.41	1.24	0.48	0.99
CTD18-FL-DNA	52,971	49,667	103	154.00	138.38	0.54	0.82	0.99
116-FL-DNA	53,709	45,664	481	705.48	823.86	2.47	0.22	0.99
118-FL-DNA	53,942	42,305	354	616.08	791.52	2.35	0.21	0.99
119-FL-DNA	52,994	48,725	360	527.50	505.50	2.39	0.18	0.99
120-FL-DNA	53,494	45,433	454	491.82	490.41	2.94	0.14	0.99
121-FL-DNA	52,877	44,351	392	505.24	503.42	2.14	0.31	0.99
122-FL-DNA	53,886	47,422	333	439.05	415.88	2.76	0.13	0.99

### Community composition of archaea

**Figure 3:**
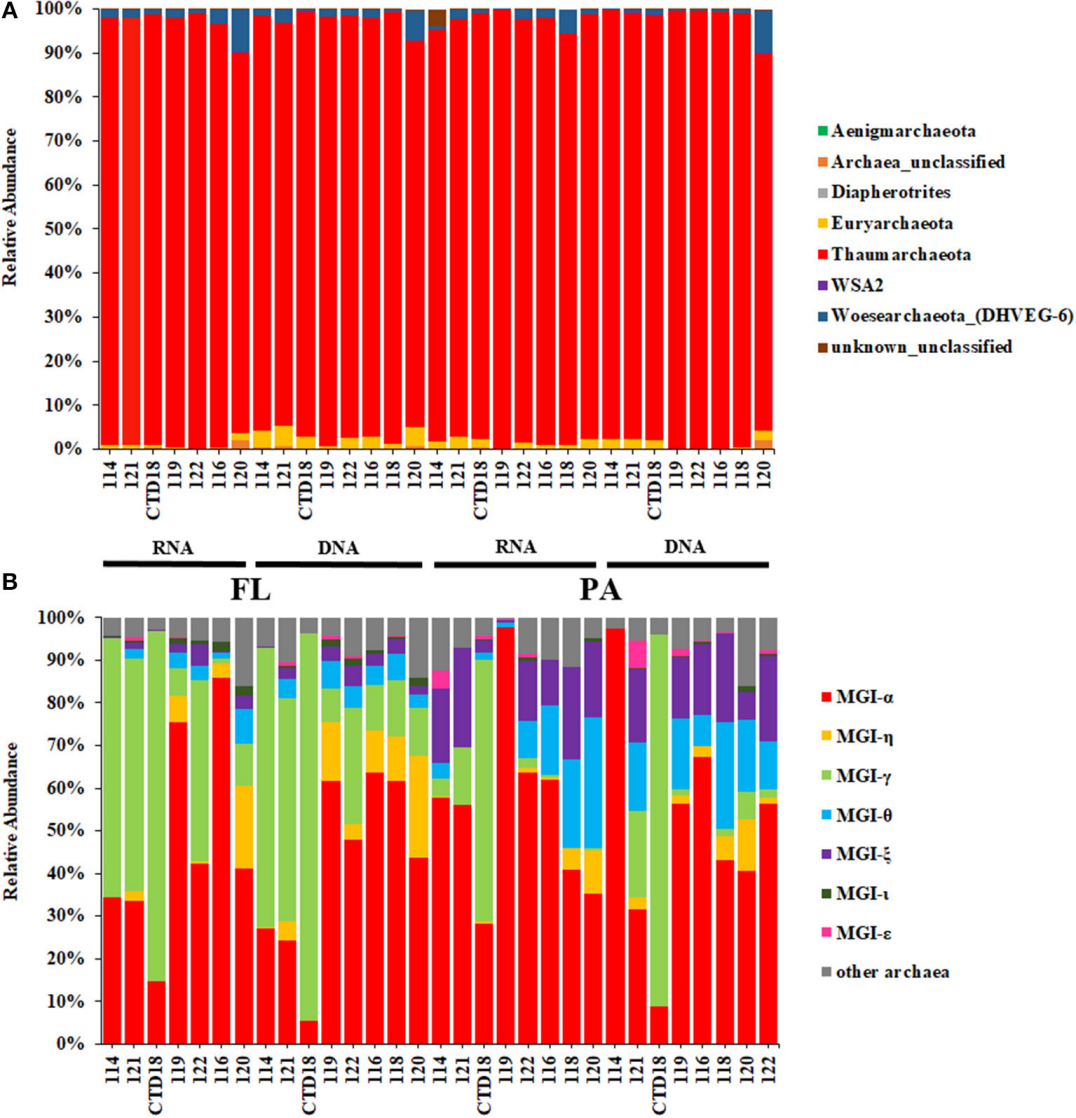
Relative abundances of archaea at the phylum level **(A)** and at the Marine Group I subgroup level **(B)**.

**Figure 4:**
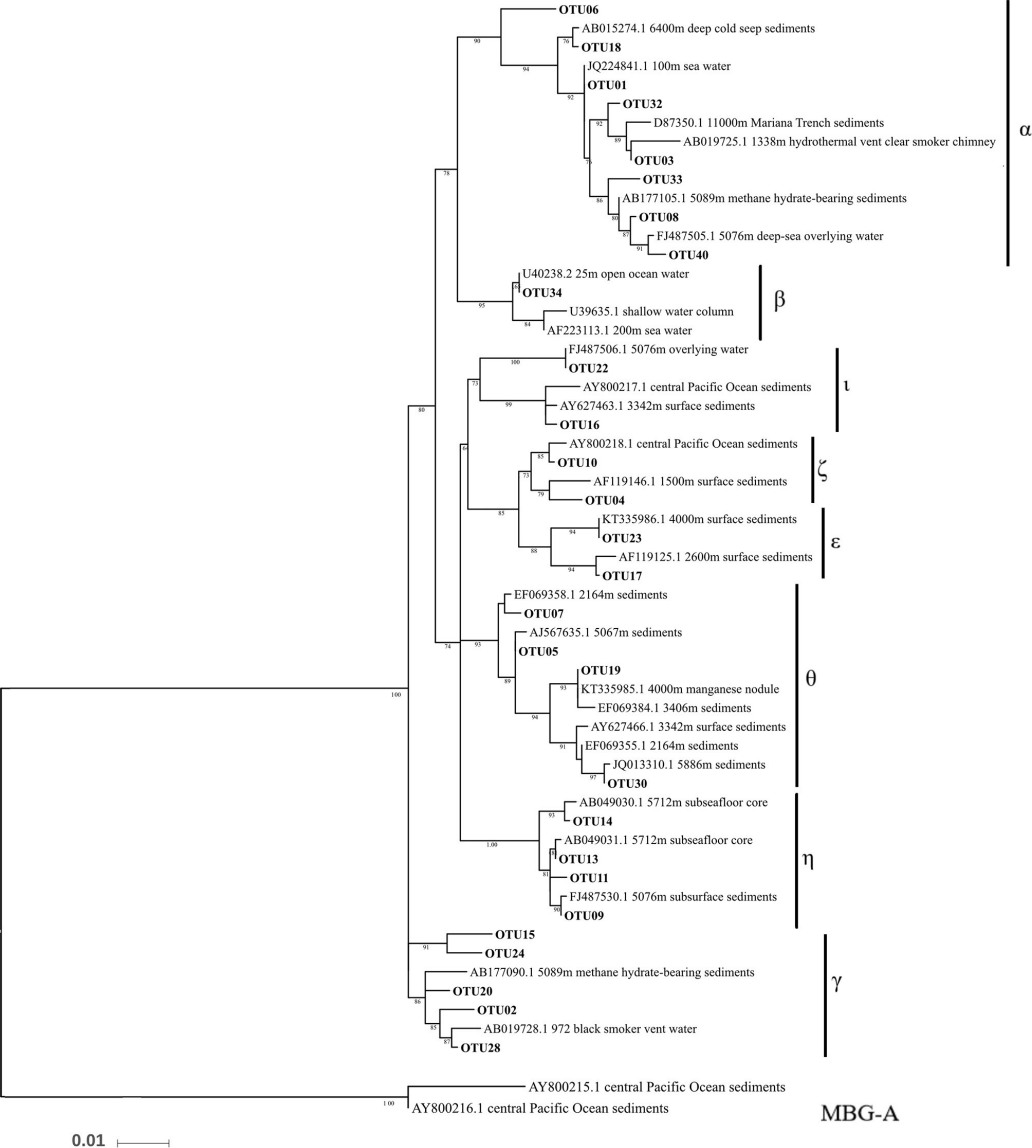
Phylogenetic maximum likelihood tree of archaeal 16S rRNA genes with Marine Group I in the top 40 OTUs. Marine Benthic Group A was placed as the outgroup.

Thaumarchaeota dominated in all the datasets, with relative abundances >90% (Figure 3A). Almost all of the Thaumarchaeota sequences were assigned to subgroups of MGI (one clade from Thaumarchaeota), including MGI-α, γ, θ, ξ, and η, as revealed by phylogenetic analysis (Figure 4). Woesearchaeota was the second most abundant taxon at the phylum level, and its relative abundance slightly increased with depth (Figure 3A).

MGI-α was dominant in all of the samples and was at a higher proportion in the samples from the southern stations than in those from the northern stations at both the DNA level and RNA level (Figure 3B). The proportion of MGI-α decreased with water depth in the PA fraction at both the DNA and RNA level. MGI-γ was more abundant in the FL fraction than in the PA fraction, whereas MGI-θ and MGI-ξ were both more abundant in the PA communities at both the DNA and RNA level. Although the proportion of MGI-θ increased with the increasing depth in the PA fraction at the RNA level, the proportion of MGI-ξ remained constant with the increasing depth in most of the samples. MGI-η was observed in all samples apart from in Dive-114, and in CTD-18, which were the control samples of this study. In addition, MGI-η was more abundant in the FL fraction than in the PA fraction.

**Figure 5:**
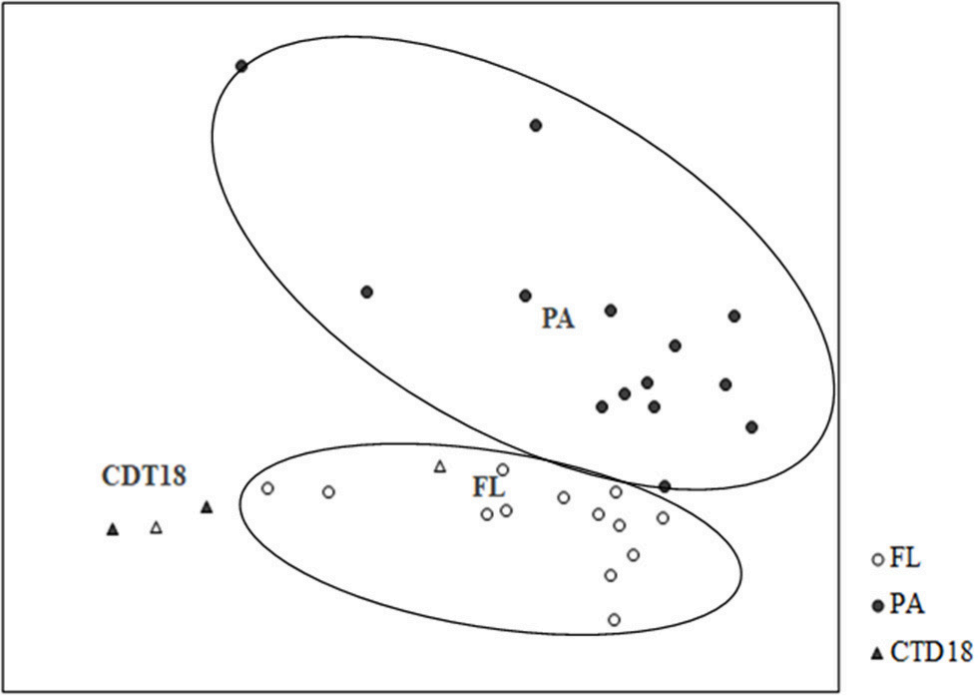
Grouping of communities according to Bray-Curtis distances using non-linear multidimensional scaling. PA and FL were separated into two groups.

The PA and FL communities at both the DNA and RNA levels were separated by UPGMA (Figure S2) and NMDS (Figure 5). Apart from the PA and FL groups, a third distinct group was found in the CTD-18 samples, which were collected from the central region of the trench. This group was clearly separated from the PA samples (*p* < 0.05) and FL samples (*p* < 0.05). In addition, the FL and PA samples were significantly different from each other (*p* < 0.01).

### Effect of environmental factors on archaeal communities

**Figure 6:**
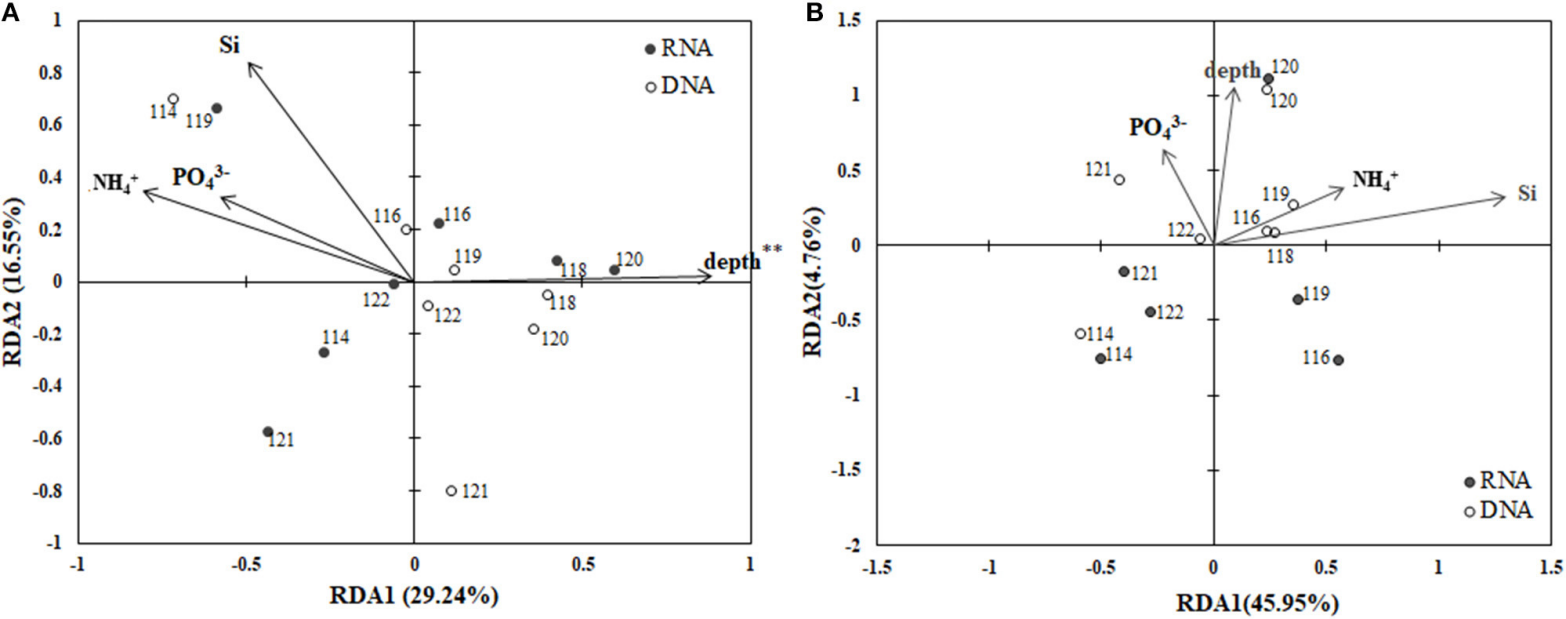
RDA ordination plots for the distributions of PA **(A)** and FL **(B)** archaeal phylogenetic groups with associated environmental parameters. **p < 0.01.

The RDA biplot (Figure 6) illustrates the relationship between the archaeal phylogenetic groups and the associated environmental factors. Data from CTD-18 were not included in this analysis due to them being significantly different from both the PA and FL datasets. In addition, the FL and PA communities were calculated separately. Water depth (*p* < 0.01) was the only environmental parameter that was shown to affect the PA communities (Figure 6A). The first and second axes contributed 29.24 and 16.55%, respectively to the total variance of these communities. In contrast, no environmental factors were identified by the RDA analysis to significantly affect the FL communities (Figure 6B).

## Discussion

### Abundance of archaeal 16S rRNA gene and gene transcript

We detected much higher gene abundances in FL archaea from the BBL in the Mariana Trench than were previously reported for water samples from similar depths in the same location (Nunoura et al., 2015). However, the archaeal 16S rRNA gene abundances in the PA fraction were lower than those reported for the surface sediments by ~2 orders of magnitude (Jørgensen et al., 2013; Danovaro et al., 2016). This difference might be due to the fact that deep-sea sediment is often disturbed by epibenthic macrofauna (Ruhl et al., 2008; Mayor et al., 2012), and bottom currents (Johnson, 1998; Siedler et al., 2004), which together promote the exchange of microbial communities between the sediment and water column. Therefore, as the BBL is a transition layer between the sediment and water column, it receives a higher level of resuspended particles from the benthic sediment than the water column above does. This results in the BBL containing lower concentrations of organic matter than the benthic sediments, but higher concentrations of organic matter than the other layers of the water column. Consequently, the BBL supports a higher abundance of FL archaea than the water column above, but a lower abundance of PA archaea than the benthic sediment.

To date, most studies on the archaeal populations in trench ecosystems have focused exclusively at the DNA level (Nunoura et al., 2015; Danovaro et al., 2016). Our study therefore represents the first investigation of the archaeal 16S rRNA gene and gene transcript in the Mariana Trench. Overall, the RNA/DNA ratios that we observed were much lower than those reported for shallow waters (Ganesh et al., 2015); we suggest that this might be due to the slow life cycles of archaea in the deep-sea (Jørgensen and Marshall, 2016). In addition, the RNA/DNA ratios were higher in the FL community than in the PA community, which suggests that most of the nutrients released from the sediments might be dissolved and that the resuspended sediment particles might exhibit a low rate of mineralization. In addition, it should be noted that the RNA extraction and cDNA quantification methods used here, have a number of constraints, which might result in underestimations at the RNA level.

### MGI dominates the archaeal communities in the BBL

Thaumarchaeota, specifically MGI, was the predominant archaeal assemblage in this study. This suggests that there is extensive nitrogen cycling and carbon fixation driven by archaea in the BBL of the Mariana Trench (Figure 3A). These results are also in agreement with the finding that there is a high ammonium concentration near to the seafloor (Table 1). Our detailed phylogenetic analysis of MGI sequences showed that the most abundant subgroup was MGI-α (including OTU01), which has also been previously reported to be dominant in deep-sea water columns (Nunoura et al., 2015), and deep-sea subsurface sediments (Massana et al., 2000; Takai et al., 2004; Durbin and Teske, 2010; Kato et al., 2013). MGI-γ was the second most abundant subgroup detected in our study and this has also been obtained previously from deep-sea water columns (Nunoura et al., 2015, 2016). Indeed, MGI-α and γ have been shown to be the most abundant archaea in deep-sea water columns, where they are dominant in hadal and abyssal oceanic regions, respectively (Nunoura et al., 2016). MGI-θ, ξ, and η were other dominant subgroups found in our study. MGI-θ and ξ are known to be mainly found in abyssal subsurface sediments (Sorensen et al., 2004; Brandt et al., 2007; Durbin and Teske, 2010), whereas MGI-η has been found in the deeper layers of deep-sea sediments (i.e., 50 cm to 150 cm below the sediment subsurface; Durbin and Teske, 2010). In addition, MGI-θ and ξ have low abundances in the subsurface sediments, whereas MGI-α is dominant to 50 cm below the sediment subsurface. MGI-η appears at depths >50 cm, and here it dominates the archaeal community, whereas in the same location MGI-α, θ, and ξ are not apparent (Durbin and Teske, 2010, 2011).

The BBL has a mixed signature, consisting of both deep water and sediment. In this study, the PA archaeal communities mostly comprised MGI-α as well as low proportions of MGI-θ and ξ; this is similar to the communities found in the subsurface sediments of the gyre abyssal plain and mid-ocean ridge (Durbin and Teske, 2010, 2011; Jorgensen et al., 2012). In contrast, the FL community not only had a high proportion of MGI-α but it was also enriched with MGI-γ, more so than MGI-θ or ξ. Similar archaeal community compositions have been found in water column samples (Nunoura et al., 2015, 2016). Interestingly, MGI-η was more abundant in the deeper stations, especially in the FL fraction, but it was absent from Dive-114 and CTD-18. CTD-18 was almost completely composed of MGI-α and γ, whereas the PA fraction of Dive-114 contained higher proportions of MGI-θ and ξ, which are two sediment MGI lineages. The major difference between these two stations (Dive-114 and CTD-18) and other stations is the sampling distance from the benthic sediment. This suggests that MGI-η might be a group unique to the deep-sea BBL and sediments, and that it might be an FL archaeal group in the deep-sea BBL. Further investigation of the deep-water preference of MGI-η is required.

Woesearchaeota are the second most abundant phylum in our samples although they account for < 10% of the taxa in all of the samples. Although no isolate was obtained, Woesearchaeota have been suggested to be heterotrophs living in oligotrophic conditions, which are in symbiosis with other archaea. This is according to genomic information extracted from other environmental samples (Castelle et al., 2015; Danovaro et al., 2016). In our study, the proportion of Woesearchaeota increased slightly as the water depth increased; this might partly be explained by the higher levels of organic matter in the deeper locations.

### Effect of environmental condition on community similarities

Over the last decade, the deep-sea microbial communities have come under intense research scrutiny (Lipp et al., 2008; Caporaso et al., 2011; Nunoura et al., 2015; Tarn et al., 2016). In this study, we assessed archaeal community heterogeneity along the Mariana Trench, and found that in the BBL, the depth of the water was the most influential factor that shaped the PA archaeal community structure, especially with regards to MGI sub-diversity (Figure 6). According to our RDA results, the archaeal community structures were significant relative to the depth. This finding is consistent with previous studies, which showed that depth is a key factor contributing to the niche separation of archaeal communities, especially the MGI subgroups (Blackburne et al., 2007; Martens-Habbena et al., 2009; Sintes et al., 2013; Nunoura et al., 2015). However, several studies suggested that rather than the depth *per se*, it was the nutrients and organic matter at the different depths, which control the archaeal community composition in abyssal regions (Lipp et al., 2008; Caporaso et al., 2011; Tarn et al., 2016).

In addition, the TOC/TN (total organic carbon/total nitrogen) ratio has also been suggested to be an influencing factor on the composition of the deep-sea archaeal communities (Luo et al., 2015). In some organic-poor and high-TOC/TN-ratio areas, abundant Thaumarchaeota of other lineages also exist. These include the Miscellaneous Crenarchaeotal Group, Marine Benthic Groups A and B, and Marine Hydrothermal Vents Groups B and C (Durbin and Teske, 2012). According to a study by Luo et al. (2017), the variation in TOC content among all of the samples was < 0.1%. Since some of our stations were adjacent to theirs, therefore, TOC was excluded as a key factor. In addition, the higher abundance of archaeal 16S gene transcripts in the FL fraction, indicated that in the BBL of the trench slopes, dissolved nutrients that are released from the deep layer sediment (Nunoura et al., 2013), such as NH4^+^, are more available to archaea, especially MGI. This suggests that any remaining organic matter during settling is unavailable and that the concentration of inorganic nutrients might be the key factor that influences the niche separation of the MGI subgroups.

Although high hydrostatic pressure is known to reduce the activity of microbial metabolism (Mendes et al., 2007; Picard and Daniel, 2013), the influence of hydrostatic pressure on microbial community composition has not been reported. As our sampling stations did not have a continuum of depths, a clear trend of archaeal community shifts with depth could not be observed. A continuous *in situ* detection of archaeal taxa in the BBL of the slopes of the Mariana Trench, or in a laboratory simulation of pressure effects on the deep-sea BBL archaeal community, might be helpful.

In our study, the effect of depth was more obvious due to the inclusion of control station CTD-18, which was located in the central region of the trench and far from the benthic sediment. In contrast, other stations were located along the slopes adjacent to the benthic sediment. It is likely that the steep slopes and narrow geomorphology of the Mariana Trench might have a condensation effect for suspended organic particles that sink from the surface of the ocean (Nunoura et al., 2015). This might consequently also influence the diversity and activity of PA archaea in the BBL of the Mariana Trench. However, due to the limited number of samples in our present study, a more comprehensive survey over a larger geographical scale is required in order to clearly define the ecological role of archaea in the BBL and their responses to environmental change.

## Conclusions

We investigated the abundance and diversity of PA and FL archaea at both the DNA and RNA levels in the BBL along the slopes of the Mariana Trench. The PA and FL archaeal communities had composition patterns resembling those of sediment and water column samples, respectively. The co-occurrence of some active subgroups of archaea, such as MGI-η, in both the PA and FL communities indicates that interaction and exchange between the sediment and overlying water body occurs in the BBL. In addition, our results revealed that 16S rRNA gene transcripts between PA and FL archaeal communities were distinct in deep-sea BBL, and we suggest that this might result from niche separation caused by diffusible nutrients from the sediment, and difficulties with the mineralisation of organic matter in particles. The low proportion of the heterotrophic Woesearchaeota along the northern and southern slopes of the deep trenches might be due to the poor availability of food/nutrient sources. These findings provide new insights into the deep-sea archaeal communities and their activities in the deep-sea BBL. Investigations into the ecological factors that shape these communities and their capacity for carbon fixation are now ongoing.

## Author contributions

HJ conceived and designed the experiments. WZ and LZ performed the experiments. WZ analyzed the data. HJ and HL contributed reagents, materials and analysis tools. WZ wrote the paper. HJ, LZ, HL, and YZ contributed writing and analysis guidance.

### Conflict of interest statement

The authors declare that the research was conducted in the absence of any commercial or financial relationships that could be construed as a potential conflict of interest.
