# Supplementary material for: Particle-Attached and Free-Living Archaeal Communities in the Benthic Boundary Layer of the Mariana Trench
Source: Front Microbiol. 2018 Nov 21;9:2821. doi: 10.3389/fmicb.2018.02821 (PMC6258811; doi:10.3389/fmicb.2018.02821)
Supplement: Supplementary file 1 [file Data_Sheet_1.docx]

**Supplementary materials**

**Figure legends:**

**Figure S1.** Venn diagram representing the overlap of OTUs among different samples. FLR: FL fraction at RNA level; FLD: FL fraction at DNA level; PAR: PA fraction at RNA level; PAD: PA fraction at DNA level.

**Figure S2.** UPGMA clustering based on square root transformed OTUs abundance from different samples at DNA (A) and RNA (B) level.


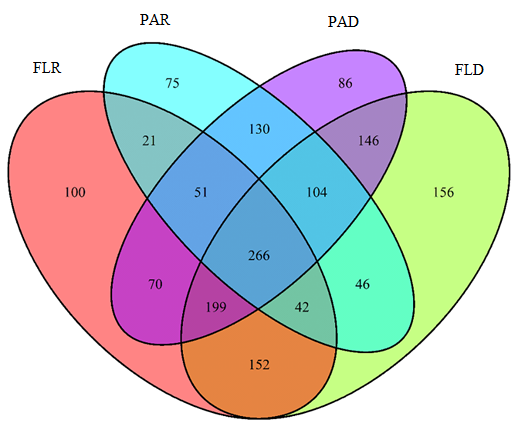


**Figure S1**


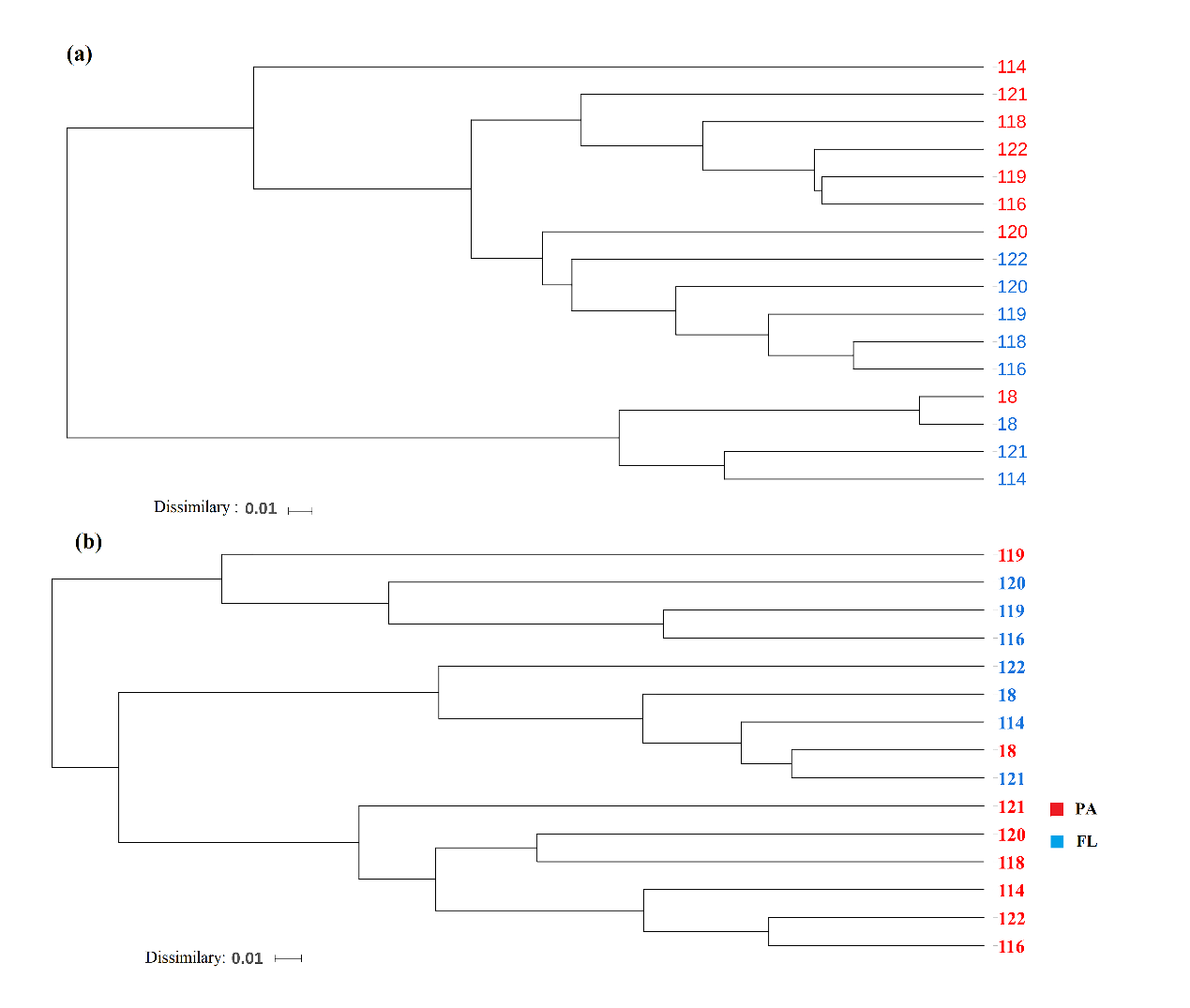


**Figure S2**
